# *C. tropicalis* promotes CRC by down-regulating tumor cell-intrinsic PD-1 receptor via autophagy

**DOI:** 10.7150/jca.79664

**Published:** 2023-06-19

**Authors:** Junxing Qu, Qianyu Chen, Ziqian Bing, Sunan Shen, Yayi Hou, Mingming Lv, Tingting Wang

**Affiliations:** 1 The State Key Laboratory of Pharmaceutical Biotechnology, Division of Immunology, Medical School, Nanjing University, Nanjing, China.; 2 Institutes of Health Central Plains, Xinxiang Medical University, Xinxiang, Henan, China.; 3 Jiangsu Key Laboratory of Molecular Medicine, Division of Immunology, Medical School, Nanjing University, Nanjing, China.; 4 Department of Breast, Women's Hospital of Nanjing Medical University, Nanjing Maternity and Child Health Care Hospital, Nanjing, 210004, China.

**Keywords:** colorectal cancer, Candida. tropicalis, PD-1 receptor, autophagy

## Abstract

**Background:** The programmed cell death 1 (PD-1) receptor is an immune checkpoint molecule that induces immune tolerance and mediates the immune escape of tumor cells. It is mainly expressed in immune cells such as T cells, B cells and monocytes. In recent years, studies have shown that tumor cell-intrinsic PD-1 plays different roles in the development of melanoma, Liver cancer and lung cancer. However, the expression and function of PD-1 in colon cancer cells has not been reported. Our previous studies have found that *Candida tropicalis* (*C. tropicalis*) can promote CRC tumor growth and chemotherapy resistance to oxaliplatin by regulating mismatch repair system. Whether *C. tropicalis* participates in the progression of CRC and immunotherapy resistance through regulating the tumor cell-intrinsic PD-1 remains to be further elucidated.

**Methods & Results:** In this study, we first found that high concentrations of *C. tropicalis* promote tumor growth in cell cultures and xenografts. In addition, we proved that colon cancer cell lines express PD-1 receptors. Knockdown of PD-1 enhanced SW480 viability in-vitro, while overexpression of PD-1 diminished cell viability. Moreover, blocking antibody against PD-1 promotes tumor growth both in SW480 cells and mice CRC xenografts in an adaptive immune-independent manner. We also demonstrated that high concentrations of *C. tropicalis* can down-regulate tumor cell-intrinsic PD-1 expression in colon cancer cells. CRC cell growth induced by *C. tropicalis* is partially offset in the presence of PD-1 overexpression. This shows that *C. tropicalis* promotes CRC progression via controlling the expression of tumor cell-intrinsic PD-1. Mechanistically, we found that *C. tropicalis* modulates the expression of PD-1 via increasing the autophagy traffic in colon cancer cells. Combining autophagy inhibitor with *C. tropicalis* treatment partly blocked the CRC tumor growth and reversed the downregulation of PD-1.

**Conclusion:** This study shows that PD-1 is a tumor suppressor in CRC. *C. tropicalis* can down-regulate tumor cell-intrinsic PD-1 expression via enhancing tumor cells autophagy levels to promote CRC progression. It may provide a new idea and mechanism for answering why the immune monoclonal antibody treatment is ineffective in cancer patients.

## Introduction

Colorectal cancer (CRC) is the most common gastrointestinal malignancy which is always accompanied by high metastasis in the United States and the CRC prognosis biomarker is still poor currently 1, 2. Numerous therapeutics including surgery, cytotoxic chemotherapy, immunotherapy, radiation, and combination strategies were applied to CRC treatments. Although immune checkpoint inhibitors represented by PD-1-blocking antibody are initially achieved in clinical trials, there are still many patients that cannot benefit from it 1, 3-5.

As one of the crucial immune checkpoint molecules, PD-1 belongs to the CD28/CTLA-4 family 6. It is mainly expressed on mature cytotoxic T lymphocytes 7, 8, and it can inhibit T cell function by binding with PD-L1 or PD-L2 ligand, which is mainly expressed on the surface of tumor cells, to induce immune tolerance and limit autoimmunity 9. In addition to being expressed in activated mature T cells, studies have shown that PD-1 as a co-inhibitory receptor is also expressed at low levels on B cells, monocytes and other immune cells 10. However, in recent years, more and more studies have shown that not only immune cells, but also tumor cells expressed PD-1. Furthermore, tumor cell-intrinsic PD-1 plays different roles in the occurrence and development of different cancers. Sonja et al. found for the first time that melanoma cell-intrinsic PD-1 receptor could promote tumor growth 11. Then Qinghai Ye 's team proved that hepatoma cell-intrinsic PD-1 induced liver tumor growth, and found that this promotion can be restrained by PD-1 checkpoint blockade in combination with mTOR inhibitors 12. Nevertheless, some other studies have shown that tumor cell-intrinsic PD-1 receptor is a tumor suppressor. PD-1 was found on lung cancer cells in 2018 for the first time. Blockade of tumor-expressed PD-1 promotes lung cancer growth 13. Shan Gao's group also reached the same conclusion. Meanwhile, they also proved that the tumor cell-intrinsic PD-1 receptor mediates the resistance of PD-1 monoclonal antibody therapy 14. At present, researches on tumor cell-intrinsic PD-1 are limited to the several cancers mentioned above. The expression of PD-1 on colon cancer cells and its roles in CRC occurrence and development have not been reported yet.

Microbial community in the human gastrointestinal tract is critical to the health and disease of the host 15. The complex interaction between the host and the gut microbiota modulates the intestinal homeostasis 16. Many studies have shown that gut microbes are involved in regulating CRC pathogenesis 17. However, most studies about symbiotic microbiota focus on bacteria, commensal fungi are rarely reported. Our previous study has demonstrated that the *C. tropicalis*, which is significantly upregulated in CRC patients and colon cancer animal models, can promote tumor progression by inducing the differentiation of MDSCs in the tumor microenvironment 18. We also found that *C. tropicalis* promoted CRC chemotherapy resistance to oxaliplatin in previous study 19. But whether this conditional pathogenic fungus is involved in the CRC via regulating tumor cell-intrinsic PD-1 receptor needs to be further clarified.

In this study, we found that colon cancer cell lines express both PD-1 and PD-L1. While the adaptive immunity is deficient, the PD-1/PD-L1 signal axis inhibits tumor growth through AKT/ERK pathway. Our study also proved that high concentrations of *C. tropicalis* promote the development of CRC via down-regulating tumor cell-intrinsic PD-1 expression. Furthermore, we found that *C. tropicalis* modulates PD-1 expression by increasing the flux of autophagy in tumor cells. Our research broadens the understanding of the effects of anti-PD-1 immunotherapy on tumor cells. Meanwhile, we clarified that tumor cell-intrinsic PD-1 is a potential target in the process of *C. tropicalis* promoting the development of CRC, it provides a new idea for the CRC therapy.

## Materials and Methods

### Reagents and Antibodies

The *C. tropicalis* strain (W4162870) was kindly supplied by Dr. Sarah L. Gaffen (University of Pittsburgh, PA). Nivolumab (A2002) was purchased from Selleck Chemicals (Houston, Texas, USA). LY294002 (#9901) was purchased from Cell Signaling Technology (Boston, MO, USA). Chloroquine (C6628) was purchased from Sigma-Aldrich (St. Louis, MO, USA). GFP-mCherry-LC3 adenoviral vectors were purchased from Beyotime Biotechnology (Shanghai, China). The following antibodies were used for western blot: anti-PD-1 (Human specific, #86163), anti-PD-1 (Mouse specific, #84651), anti-p-Akt (Ser473, #4060), anti-p-p44/42 MAPK (Erk1/2) (Thr202/Tyr204, #4370), anti-LC3B (#3868), anti-ATG7 (#8558), anti-PD-L1 (#13684) and anti-β-Actin (#3700) were purchased from Cell Signaling Technology (Boston, MO, USA). Anti-PD-1 (# 66240-1-Ig), anti-P62/SQSTM1 (18420-1-AP) were purchased from Proteintech (Wuhan, Hubei, P.R.C). The following antibodies were used for flow cytometry: anti-human PD-1-PE (329906) was purchased from Biolegend (San Diego, CA, USA). The following antibodies were used for IHC staining: anti-PCNA (#13110) was purchased from Cell Signaling Technology (Boston, MO, USA). CCK8 Kit (CK04) was purchased from Dojindo Laboratories (Japan). Cell Trace CFSE Cell Proliferation Kit (C34554) was purchased from Invitrogen (ThermoFisher, Shanghai, China)). BCA kit was purchased from Beyotime Biotechnology (Shanghai, China).

### Mice and Animal Models

To establish CRC xenograft mouse model, four-week-old male BALB/c nude mice were purchased from Model Animal Research Center of Nanjing University and bred in independent ventilation system under specific pathogen-free conditions. 1 × 10^ 7^ SW480 cells were administrated by subcutaneous inoculation into the right axilla of each mouse. Mice were randomly divided into different groups a week after injection (n=4 each group). *C. tropicalis* was injected intratumorally at multiple points, twice per week for three weeks. Nivolumab and IgG control antibodies were given by intraperitoneal injection in separately every 2 d for 3 wks. Chloroquine (CQ) was administrated by intraperitoneal injection, twice per week for three weeks. The length and width of the tumors were measured two times a week or every 3-4 days with calipers. Tumor volumes were assayed by calculating the formula (A × B ^2^)/2, where A and B are the long and short dimensions, respectively. After three weeks, all mice were sacrificed and tumors were collected and weighed. All animal operations were performed in compliance with the “National Institutes of Health Guidelines for the Care and Use of Laboratory Animals” and were approved by the Institutional Animal Care and Use Committee of Nanjing University Medical School.

### Fungi Strain and Growth Conditions

*C. tropicalis* was expanded overnight on a shaker at 220 rpm/min in liquid Sabouraud Dextrose Medium at 30 °C.

### Immunohistochemistry

Subcutaneous tumor tissues were obtained from mice under different treatments. Then the fresh tumor sections were fixed in 4% paraformaldehyde (PFA). The tumor samples were gradually dehydrated and then embedded in paraffin. The tumor sections in the paraffin were cut into 5μm in thickness and then deparaffinized and incubated in citrate buffer at 95℃ for 40 min for antigen retrieval, and then incubated overnight at 4℃with primary antibody (PCNA,1:200, CST, USA). After three washes, tissue slices were incubated with biotinylated anti-mouse IgG (1:200 dilution, CST, USA) for 1 hour at RT and then washed three times.Then streptavidinhorseradish peroxidase conjugates (Vector Laboratories, USA) were added to the slices and incubated for 45 min. After washing three times, DAB solution (Vector Laboratories, USA) was added and the slides were counterstained with hematoxylin.Proliferation index which refers to positive cells/tumor cells with brown staining were counted in 10 consecutive areas.

### RNA Extraction and Quantitative RT-PCR

CRC cells 'total RNA including SW480 cells, CT26 cells and MC38 cells were isolated using TRIzol reagent. Then the reverse transcription was performed with1 pg-1 µg of total RNA in a 20 µL system according to the manufacturer's instructions (Vazyme company, China).

qRT-PCR amplification was performed on an Applied Biosystem Viia 7 quantitative PCR system (Applied Biosystems, Foster City, CA) using SYBR Green with an ABI Step One Plus system (Life Technologies). The primers used for qRT-PCR amplification are listed in Table 1. PCR system consisted of 45 cycles of denaturation at 95 °C for 2 min, annealing at 60 °C for 30s, and extension at 72 °C for 30s. All reactions were run in triplicate. The gene expression levels were normalized to the housekeeping gene, β-actin and calculated using 2^-ΔΔCt^ method.

### Western Blot

Tumor tissues were homogenized with 3 steel beads in lysis buffer (300ul lysis buffer was added in 30mg tumors). Then the mixture was centrifuged at 14,000 × g for 10 min and the proteins were extracted. As for cell samples, 200ul lysis buffer was added in 1*10^6^ cells. Lysates were put on ice for 20 min and then centrifuged at 14,000 × g for 10 min. Subsequently, protein concentration was determined using BCA kit (Beyotime Biotechnology). According to the BCA kit results, 30 µg of protein per lane was separated on 10% and 15% polyacrylamide gels and transferred on to polyvinylidene difluoride membranes (Millipore, Billerica, MA, USA). Membranes were blocked with 5% bovine serum albumin (BSA) dissolved in Tris-buffered saline containing 0.1% Tween 20, and then the membranes were incubated with specific antibodies in a proper concentration at 4℃overnight. After incubating the secondary antibody on the next day, use the Imaging System to expose. The values were normalized to the β-actin intensity levels.

### Flow cytometric analysis

Tumor tissues were cut into pieces and digested with collagenase and DNase in RPMI 1640 medium, then the mixture was passed through a 200-mesh sieve to obtain single cell suspension. After cell counting, 1*10^6^ cells were divided into flow tubes and stained with anti-PD-1-PE antibody (Biolegend, San Diego, CA, US). The cell mixture was detected by a FACS Calibur flow cytometer (BD Bioscience) and data were analyzed using FlowJo software (TreeStar, Ashland, OR). The gating strategy is based on the blank control.

### Cell culture

Colorectal cancer cell line SW480, CT26 and MC38 were obtained from the Type Culture Collection of the Chinese Academy of Sciences (Shanghai, China) and were cultured in RPMI-1640 medium (GIBCO, Carlsbad, CA) supplemented with 10% fetal bovine serum (FBS), 1% (100 U/mL penicillin and 100 ug/mL streptomycin) at 37 °C in a humidified 5% CO 2 atmosphere. CT26 and MC38 cells were seeded in cell plates to detect the PD-1 expression. SW480 cells were seeded onto different types of plates and treated under different conditions for further experiments when the cell density reached ~80%.

### Cell viability assay

To assess the cell viability under different conditions, CCK-8 assays were used according to the manufacturer's instructions (Dojindo Laboratories, Japan). SW480 cells were seeded onto 96-well plates at a concentration of ~4 × 10 ^4^ cells/well with 100μL culture medium. After different stimulations, the 10μL of CCK-8 solution plus 90ul RPMI 1640 medium were added to the cells at specific time points and cells were incubated for 2hrs at 37 °C. OD values were detected using Microplate reader (Biotech, USA).

### Immunofluorescence

Cultured cells were seeded on glass coverslips in six-well plates and transfected with GFP-mCherry-LC3 adenoviral vectors when the cells reach 50%-70% confluence. After three PBS washes, the samples were fixed with 4% paraformaldehyde. Then the nuclei were stained by DAPI. Slides were visualized using a confocal laser scanning microscope (FV3000, Olympus).

The principle of the assay is based on different pH stability of green and red fluorescent proteins. The fluorescent of GFP could be quenched under the acidic condition in the lysosome, and the mCherry fluorescent was not influenced under the acidic condition. All adenoviral infection was performed according to the manufacturer's instructions.

### CellTrace CFSE Cell Proliferation Assay

To assess the cell proliferation under different conditions, CFSE assays were performed according to the manufacturer's protocols. SW480 cells were seeded onto 6-well plates at a concentration of ~1 × 10^6^ cells/well and labeled with CellTrace CFSE Cell Proliferation Kit according to the manufacturer's instructions. Cells were treated with different stimulations for certain time and analyzed using a FACS Calibur flow cytometer (BD Bioscience) and data were analyzed using FlowJo software (TreeStar, Ashland, OR).

### siRNA Knockdown Transfection

siRNA was transfected according to the manufacturer's instructions (Ruibo company, China). The concentration of si*PDCD1* and si*PDCD1LG1* used in the study were all 50nM. The *PDCD1* siRNA target sequence is GCTTCGTGCTAAACTGGTA and GTATGCCACCATTGTCTTT, and the *PDCD1LG1* siRNA target sequence is GCTGTCTTTATATTCATGA and CCATACAACAAAATCAACC. The target sequence of non-coding (NC) siRNA was a random sequence with no biological effects.

### Statistical Analysis

Statistical analysis was conducted in GraphPad Prism 7. A two-tailed Student's t-test was employed to analyze statistical significance between two groups. Data are shown as means ± SD. P < 0.05 was considered to be statistically significant.

## Results

### High concentrations of *C. tropicalis* promote CRC tumor growth

**Figure 1:**
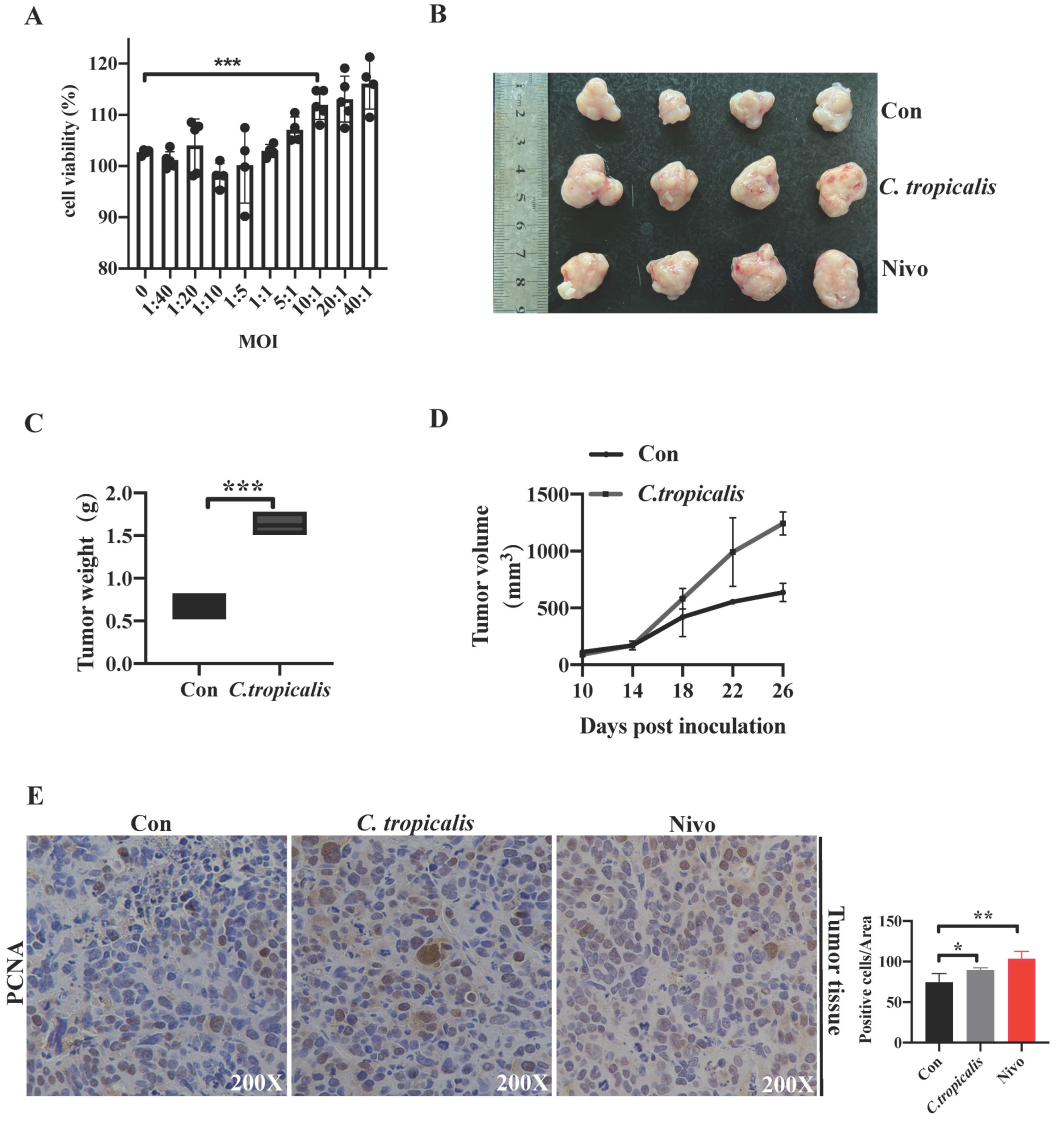
** High concentrations of *C. tropicalis* promote CRC tumor growth.** (A) Different MOI of *C. tropicalis* were used in the co-culture with SW480 cells. Cell viability was detected using CCK8. (B-F) *C. tropicalis* was injected intratumorally or not into the SW480 cells-induced xenograft CRC mouse model, n=4/group. Tumors were acquired. Representative data of tumors are shown in the first two lines (B). Statistical analysis of tumor volumes and weights (C-D). Representative images of immunohistochemical staining of tumors in different groups for PCNA. Positive cells of PCNA were counted using Image-Pro Plus software 6.0 (E). Data with error bars are represented as mean ± SD. Each panel is a representative experiment of at least three independent biological replicates. *p < 0.05, **p < 0.01 and ***p<0.001 as determined by unpaired Student's t test.

Our previous study has demonstrated that although *C. tropicalis* (multiplicity of infection, MOI = 1) itself had no effect on proliferation, migration, and apoptosis in SW480 cells, high dose of *C. tropicalis* promoted cells viability 19. To confirm the potential role of high concentrations of *C. tropicalis* on CRC tumor growth further, different MOI of *C. tropicalis* were used in the co-culture with SW480 cells. We compared cell viability among the control SW480 cells and the *C. tropicalis*-cocultured SW480 cells. High doses of *C. tropicalis* coculture resulted in increased cell viability and the facilitation is peak when MOI=10 (Figure 1A), so all next experiments were treated upon MOI=10 of *C. tropicalis*. Then we used CFSE assay to further validate the role of this dose of *C. tropicalis* on CRC cells. We found that *C. tropicalis* in MOI=10 significantly promoted cell proliferation compared to the control SW480 cells (Figure 5E). We also determined the effect of high dose of *C. tropicalis* on CRC *in vivo*. *C. tropicalis* were injected intratumorally into the SW480 cells-induced xenograft CRC mouse model. We found *C. tropicalis* treatment promoted tumor growth, presenting as increased tumor volume and tumor weight (Figure 1B-D). In consistence with that, IHC staining showed more PCNA positive cells in tumors tissues from co-treatment mice than those from control mice (Figure 1E). These results suggested that *C. tropicalis* promotes CRC tumor growth in both xenograft CRC mice and SW480 cancer cells.

### PD-1 is expressed in CRC cell lines

We knew that many studies have focused on the expression and function of PD-1 receptor on tumor cells rather than active T cells in tumor microenvironments 11-14. To investigate whether PD-1 is expressed on CRC tumor cells, our query of the database and other articles both pointed out that PD-1, encoded by *PDCD1* gene, is widely expressed in 32 cancer tissues and pure cancer cell lines including CRC 14.

**Figure 2:**
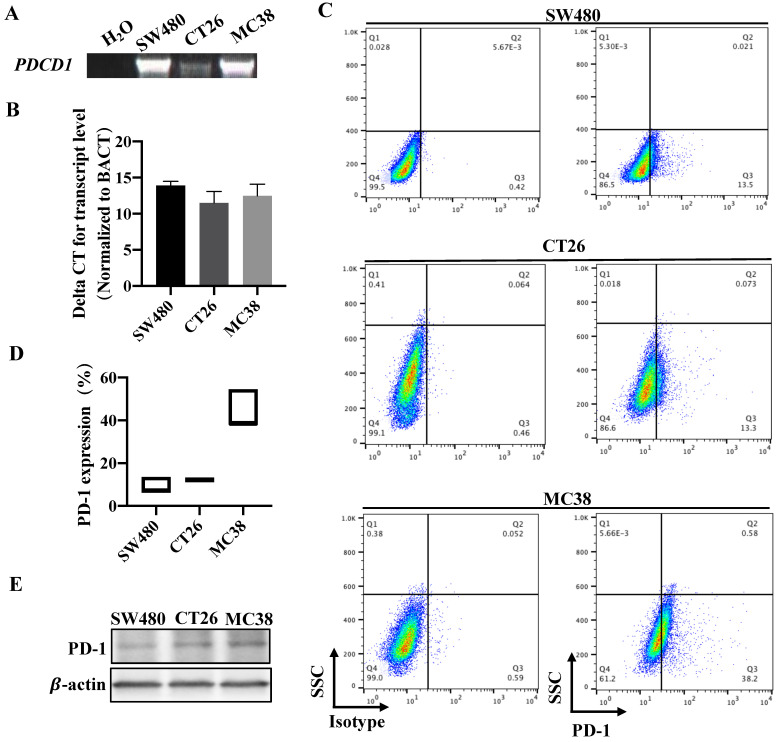
** PD-1 is expressed in CRC cell lines.** (A-C) RT-PCR, qRT-PCR expression analysis of *PDCD1* mRNA levels and western blot for PD-1 protein expression in CRC cancer cell lines. (D) Representative flow cytometry plots (Left) and percentages (Right) of PD-1 surface protein expression on CRC cancer cell lines (n=3 independent experiments, respectively). Data with error bars are represented as mean ± SD. Each panel is a representative experiment of at least three independent biological replicates. *p < 0.05, **p < 0.01 and ***p<0.001 as determined by unpaired Student's t test.

To verify this consequence, we next detected the expression of *PDCD1* in different CRC cell lines that derived from human or mice. RT-PCR and qRT-PCR all confirmed that *PDCD1* mRNA was expressed in our examined CRC cell lines (Figure 2A-B). Western blot also proved that PD-1 was expressed by CRC cell lines (Figure 2C). Furthermore, we used flow cytometry, the results revealed that PD-1 was expressed on the surface of all examined CRC cells (Figure 2D). We also tested the levels of PD-L1 expression in CRC tumor cells. qRT-PCR showed that *PDCD1LG1* mRNA was expressed in SW480 cells (Figure S1A). Overall, these data demonstrated that PD-1 was expressed in a subpopulation of CRC cell lines.

### PD-1 inhibits tumor cell growth and activation of AKT and ERK1/2

**Figure 3:**
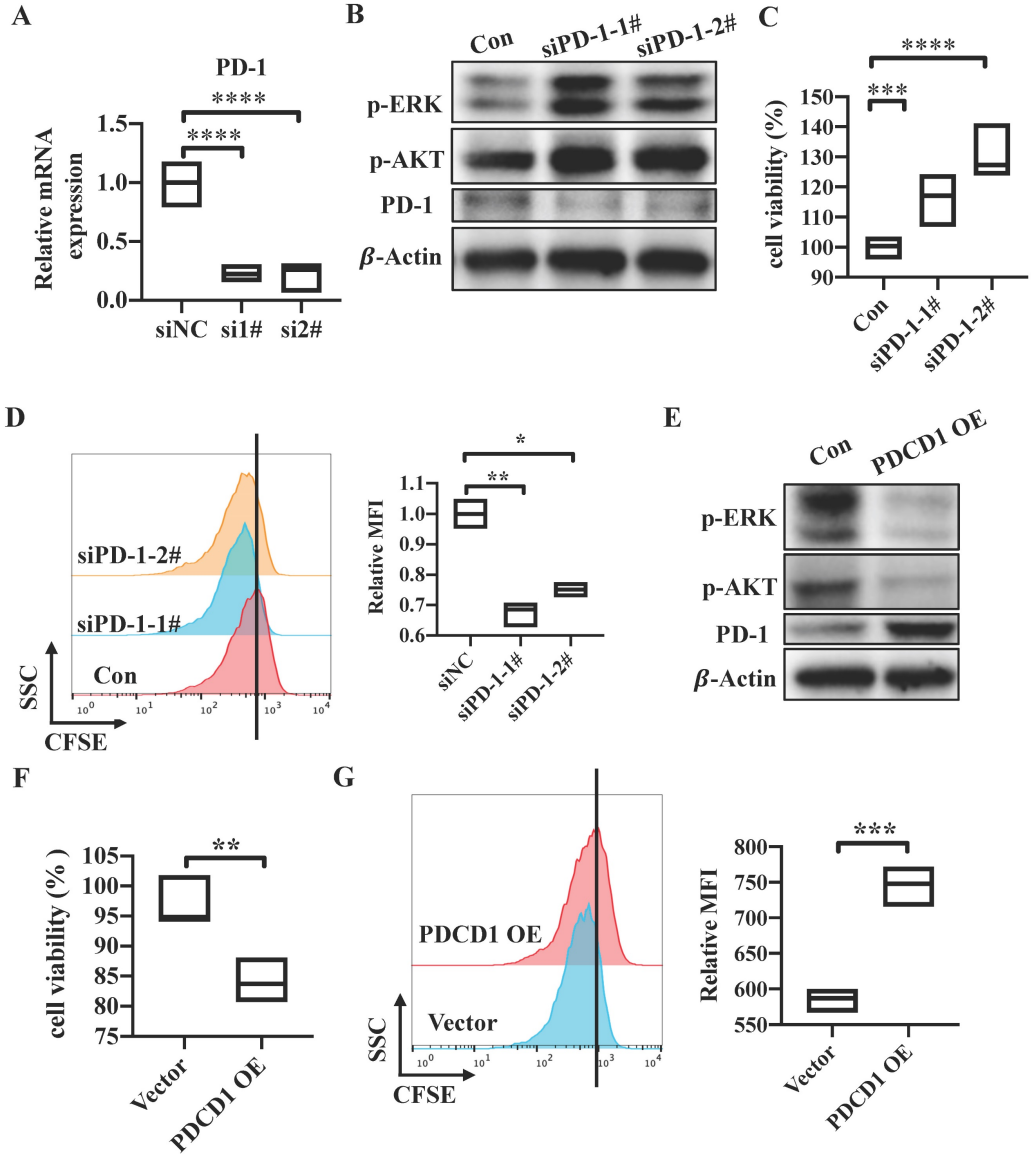
** PD-1 inhibits tumor cell growth and activation of AKT and ERK1/2.** (A-D) *PDCD1* was silenced by siRNA in SW480 cells. The knockdown function of *PDCD1* siRNA was verified by testing mRNA (A) and protein levels of PD-1 (D). CCK8 was used to detect the cell viability (B). Cell proliferation was determined by CFSE assay, quantification data was shown as MFI (n=3) (C). Western blot analysis of the indicated proteins in cells transfected with the si*PDCD1* (D). (E-G) *PDCD1* was overexpressed in SW480 cells transfected with the indicated plasmid. Western blot was used to detect the changes of PD-1 levels after transfection (E). Cell viability was determined by CCK8 (F). CFSE assay was used to detect cell proliferation (G). Data with error bars are represented as mean ± SD. Each panel is a representative experiment of at least three independent biological replicates. *p < 0.05, **p < 0.01 and ***p<0.001 as determined by unpaired Student's t test.

To explore the potential role of tumor cell-intrinsic PD-1 receptor in CRC tumor cells, we knocked down PDCD1 expression by using a short interfering RNA (siRNA) which target the sequence of *PDCD1* in SW480 cells. The mRNA and protein levels were all significantly reduced in PD-1-knockdown cells compared to control SW480 cells (Figure 3A, 3D). Cells transfected with si*PDCD1* showed increased cell viability and proliferation (Figure 3B-C). Previous study demonstrated that PD-1 led to immune tolerance mainly via modulation of downstream signals including phosphatidylinositol 3-kinase (PI3K)/AKT, MAPK/ ERK1/2 and mammalian target of rapamycin (mTOR) pathways in active cytotoxic T lymphocytes 9. Activation of AKT and ERK1/2 pathway was considered as a marker of tumor cell growth. Also, Shan Gao's research has showed that tumor cell-intrinsic PD-1 deregulated the AKT and ERK1/2 signaling pathways rather than mTOR in NSCLC cell lines 14. So, we verified if AKT and ERK1/2 signals are involved in the downstream pathways of tumor cell-intrinsic PD-1. PD-1 silencing resulted in increased levels of p-AKT and p-ERK1/2 compared with control SW480 cells (Figure 3D). Further, *PDCD1*-expressing plasmid was transfected into SW480 cells. As shown in western blot, PD-1 expression was increased in protein levels (Figure 3E). On the contrary, PD-1 overexpression resulted in decreased cell viability and proliferation (Figure 3F-G). In addition, we found that the levels of p-AKT and p-ERK1/2 were reduced in *PDCD1*-overexpressed SW480 cells (Figure 3E). We all knew that the reason why PD-1 signaling worked in T cells is the engagements of its ligands, PD-L1 or PD-L2, which are mainly expressed on cancer cells 20. As shown in Result 2, we have proved that PD-L1 is expressed in SW480 cells. So, we explored whether PD-L1 functions similarly to that of tumor cell-intrinsic PD-1. PD-L1 was knocked down by si*PDCD1LG1* in SW480 cells. The silencing efficiency was measured. mRNA and protein levels were all reduced in knockdown cells (Figure S1B-C). We found that PD-L1 silencing also resulted in enhanced cell viability and proliferation compared to control SW480 cells (Figure S1D-E). Correspondingly, PD-L1-depleted cells showed increased levels of p-AKT and p-ERK1/2 (Figure S1F). To sum up, CRC cell-intrinsic PD-1 inhibits tumor cell growth and the activation of AKT and ERK1/2 signals.

### PD-1 depletion enhances Tumorigenicity *In vitro* and *In vivo*

**Figure 4:**
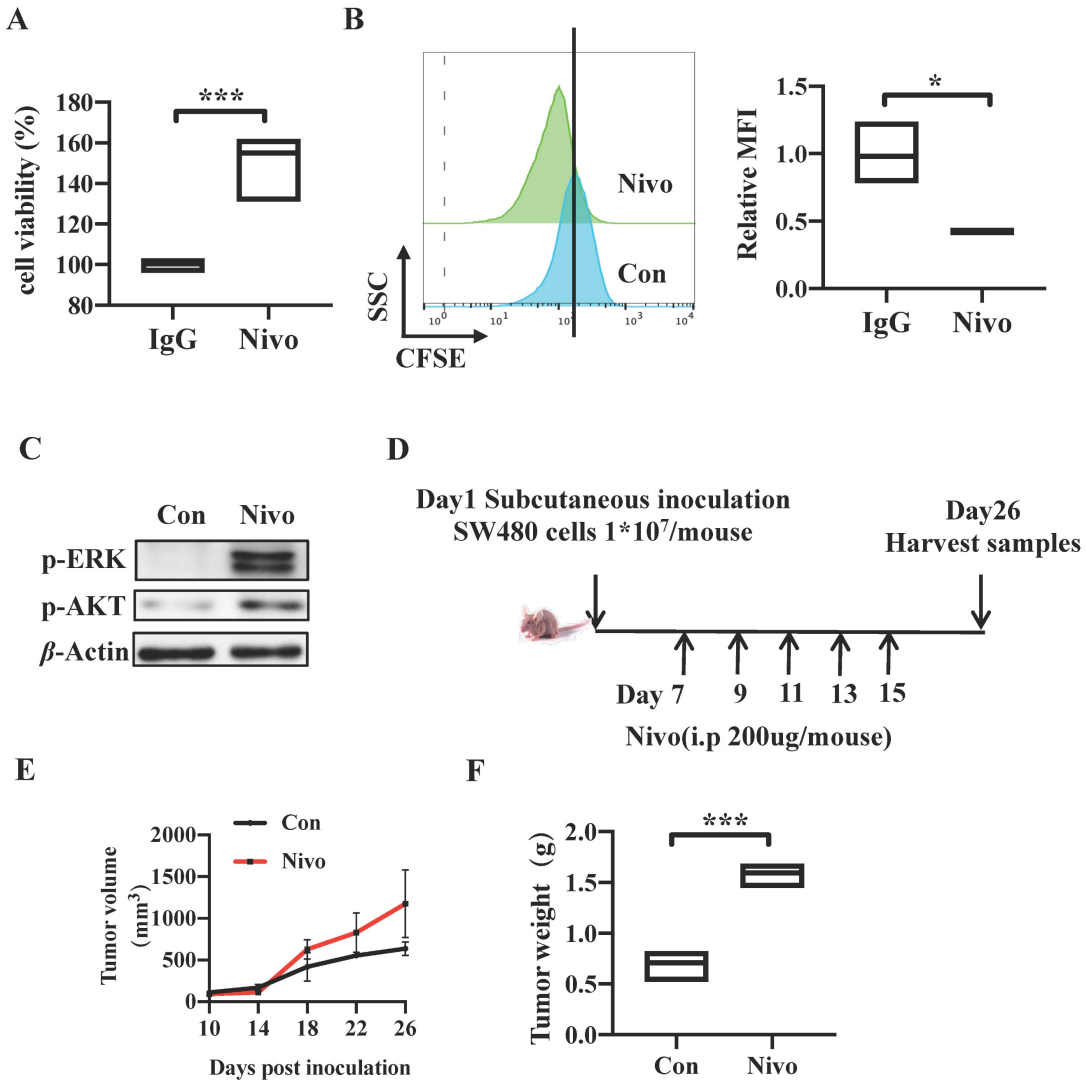
** PD-1 depletion enhances Tumorigenicity *In vitro* and *In vivo*.** (A-C) SW480 cells were treated with anti-PD-1 antibody nivolumab (100 μ g/mL) to deplete cell-intrinsic PD-1. Cell viability was detected by CCK8 (A). Representative CFSE assay assessing the relative cell proliferation. Quantification data was shown as MFI (n = 3) (B). Western blot analysis of p-AKT and p-ERK protein expression (C). (D-G) Nivolumab were injected intraperitoneally into the SW480 cells-induced xenograft CRC mouse model or not, n>=3/group. Tumors were acquired. Detailed treatment in mouse CRC xenograft model (D). Quantification of tumor size (E) and tumor weight mass (F) of the experiments. Data with error bars are represented as mean ± SD. Each panel is a representative experiment of at least three independent biological replicates. *p < 0.05, **p < 0.01 and ***p<0.001 as determined by unpaired Student's t test.

We have determined that CRC cell-intrinsic PD-1 inhibits tumor progression by knocking down and overexpressing *PDCD1*. To further explore the role of clinical drug targeting PD-1 on the genetic manipulation of PD-1, we firstly treated SW480 cells with nivolumab antibody targeting PD-1 to block the tumor cell-intrinsic PD-1 for 48h. The Cell Counting Kit-8 (CCK8) and CFSE assay results indicated that nivolumab-treated cells exhibited increased cell viability and proliferation (Figure 4A-B). Similarly, cells treated with anti-PD-1 monoclonal antibody revealed increased levels of p-AKT and p-ERK compared to the control SW480 cells (Figure 4C). In addition, to validate the effects of nivolumab *in vivo*, we established a xenograft CRC model induced by SW480 cells in nude mice and nivolumab or IgG control antibodies were injected intraperitoneally in separately every 2 d for 3 weeks (Figure 4D). Consistently, anti-PD-1 antibody treatment increased tumor volume and weight markedly in comparison with control group (Figure 1C, 4E-F). We also found that IHC staining showed more PCNA positive cells in tumors tissues from nivolumab-treatment mice than those from control mice (Figure 1E). In conclusion, these results suggested that clinical monoclonal antibody nivolumab targeting PD-1 plays the role in PD-1 gene regulation, and they also proved PD-1 depletion by nivolumab treatment promotes CRC tumor growth *in vitro* and *in vivo*.

### *C. tropicalis* promotes CRC tumor growth via down-regulating the PD-1 expression

**Figure 5:**
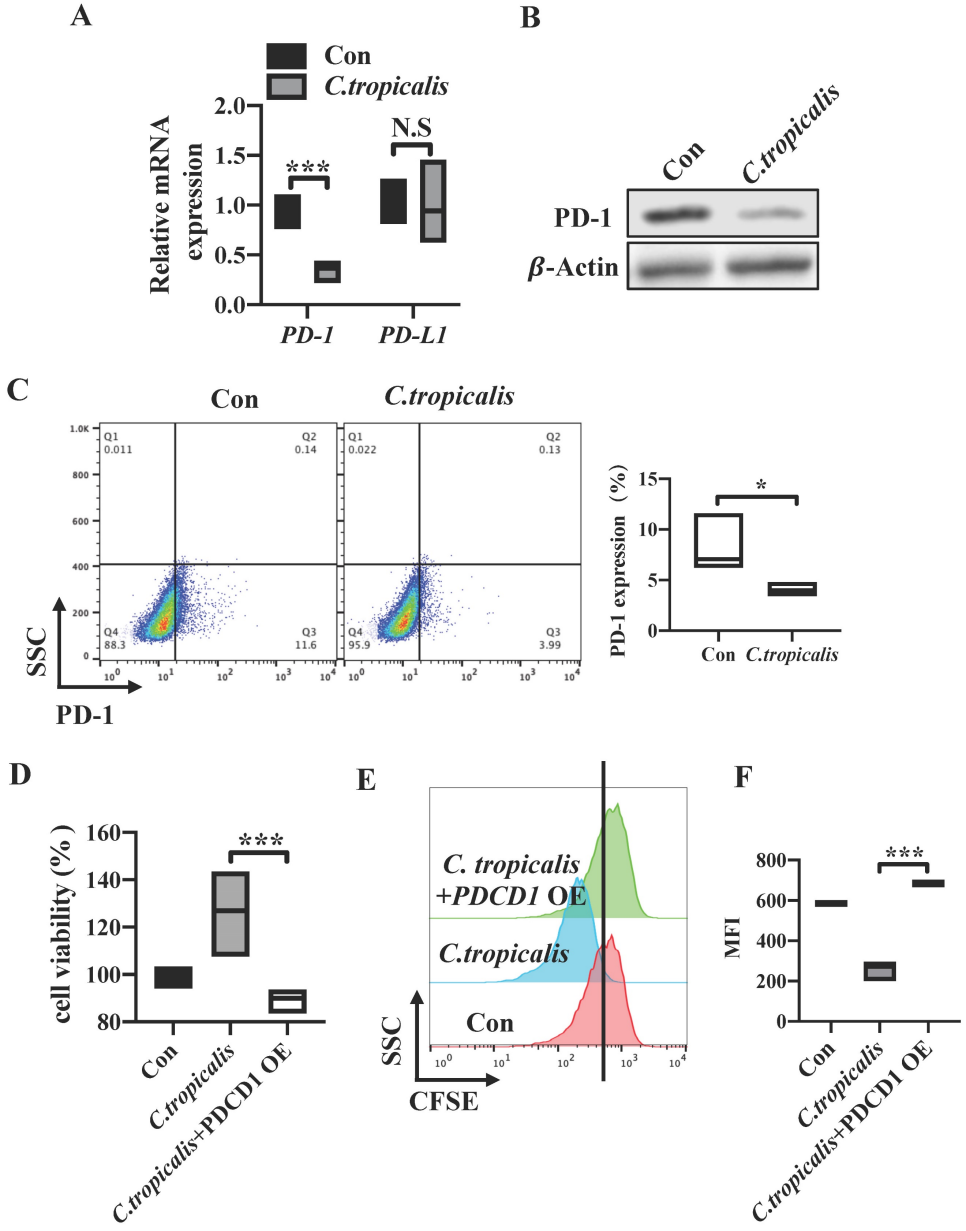
***C. tropicalis* promotes CRC tumor growth via down-regulating the PD-1 expression.** (A) PD-1 and PD-L1 mRNA expression were detected by qRT-PCR in SW480 cells cocultured with *C. tropicalis*. (B) Western blot was used to determine the changes of PD-1 protein expression after coculture with *C. tropicalis* in SW480 cells. (C) Representative flow cytometry plots and percentages of PD-1 surface protein expression in *C. tropicalis*-cocultured SW480 cells. (D-F) *PDCD1* was overexpressed in the presence or absence of *C. tropicalis* in SW480 cells. Cell viability was determined using CCK8 (D). Representative CFSE assay assessing the relative cell proliferation, quantification data was shown as MFI (n = 3) (E-F). Data with error bars are represented as mean ± SD. Each panel is a representative experiment of at least three independent biological replicates. *p < 0.05, **p < 0.01 and ***p<0.001 as determined by unpaired Student's t test.

**Figure 6:**
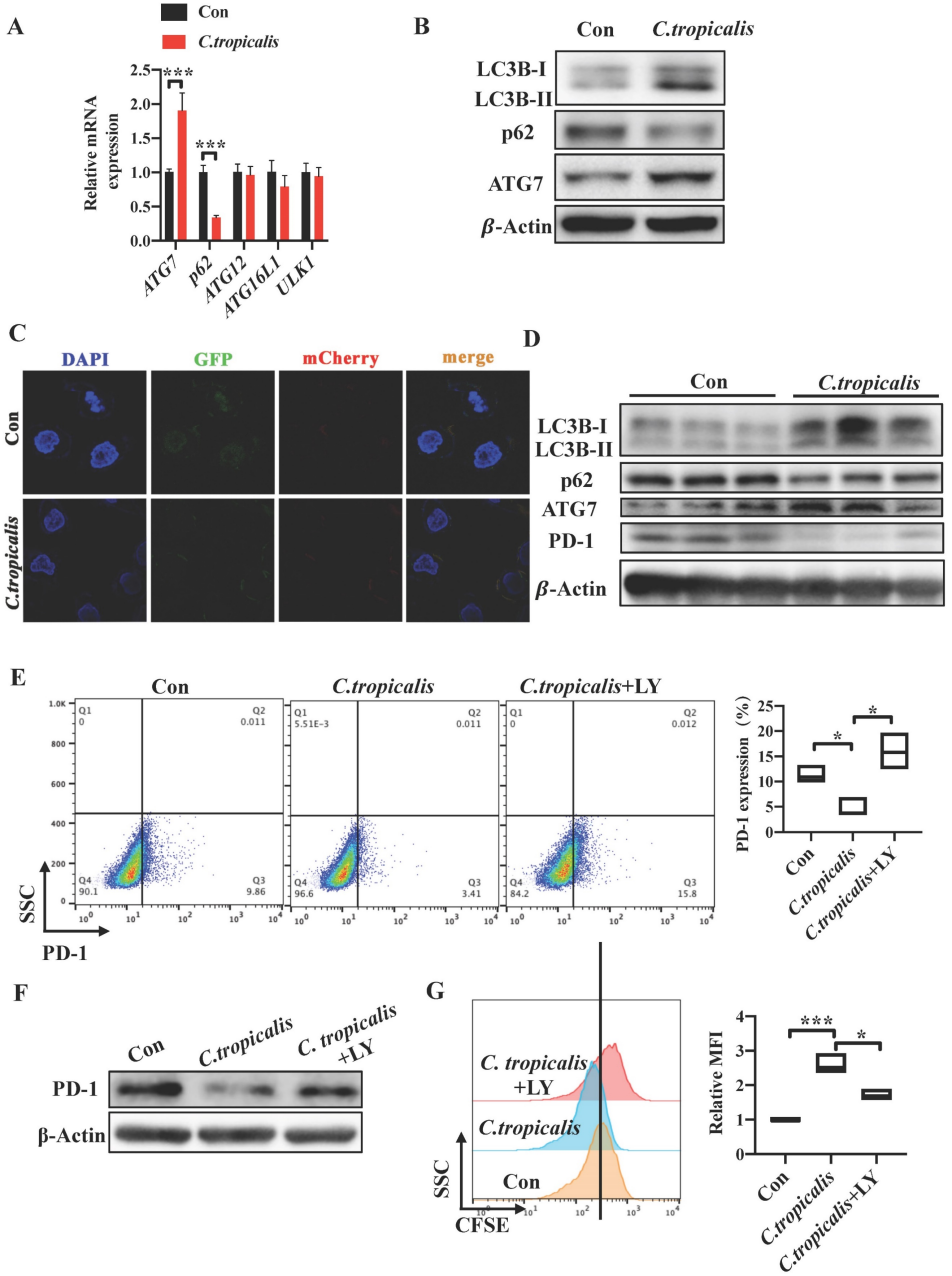

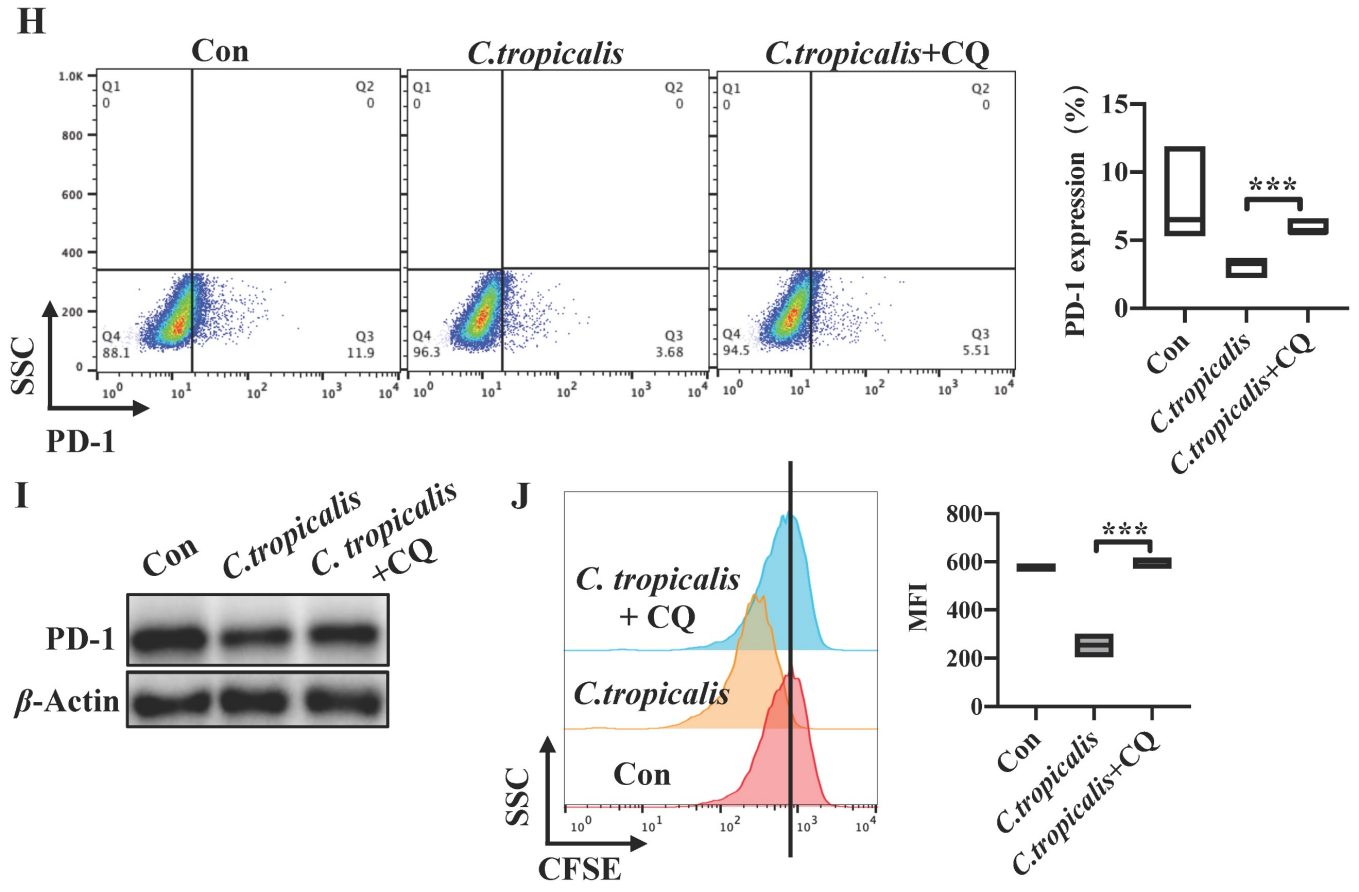
** Induction of autophagy by *C. tropicalis* mediated the regulation of PD-1 in SW480 cells.** (A) qRT-PCR was performed to detect the mRNA expression of vital proteins participated in autophagy in SW480 cells treated with* C. tropicalis*. (B) Western blot was used on autophagy element protein expression. (C) SW480 cells that stably expressed GFP-mCherry-LC3 fusion protein were co-cultured with *C. tropicalis*. Confocal microscopic analysis was shown. Bar scale, 5 µm. (D) The expression of autophagy elements indicated in (B) were determined using western blot in tumor tissues from *C. tropicalis-*treated mice. (E-G) SW480 cells were cocultured with *C. tropicalis* in the presence of LY294002, an inhibitor of autophagy. Representative flow cytometry plots and analysis of PD-1 surface protein expression (E). Western blot was performed to detect the PD-1 protein expression changes (F). Cell proliferation was determined using CFSE (G). (H-J) SW480 cells were cocultured with *C. tropicalis* in the presence of CQ, another autophagy inhibitor. PD-1 protein expression was detected by flow cytometry (H) and western blot (I). Representative CFSE assay assessing the relative cell proliferation. Quantification data was shown as MFI (n = 3) (J). Data with error bars are represented as mean ± SD. Each panel is a representative experiment of at least three independent biological replicates. *p < 0.05, **p < 0.01 and ***p<0.001 as determined by unpaired Student's t test.

We have proved that high dose of *C. tropicalis* promotes CRC tumor growth and CRC tumor cell-intrinsic PD-1 is a potential tumor suppressor. To further investigate whether tumor cell-intrinsic PD-1 is relevant to the CRC progression induced by *C. tropicalis.* Firstly, we examined the levels of PD-1 in SW480 cells upon *C. tropicalis* stimulation. We found that mRNA expression of *PDCD1* was down-regulated in *C. tropicalis-*cocultured group compared to control SW480 cells (Figure 5A). Similar result was found in the PD-1 protein levels (Figure 5B-C). We also detected PD-1 expression in CRC mouse model and found that PD-1 expression was inhibited in tumor tissues from *C. tropicalis*-treated CRC mice (Figure 6D). Conversely, the mRNA expression of *PDCDL1* was unchanged in the SW480 cells treated with *C. tropicalis* (Figure 5A). Based on these results, we came to the conclusion that PD-1 is connected with the CRC promotion caused by *C. tropicalis* instead of PD-L1. Then, to illustrate further whether the mechanism by which *C. tropicalis* promotes CRC progression is mediated by tumor cell-intrinsic PD-1, we treated *PDCD1*-overexpressed SW480 cells with *C. tropicalis*. The results showed that *C. tropicalis* still promoted tumor growth, presenting as increased tumor cell viability and proliferation. However, *PDCD1* overexpression significantly blocked the promotion of *C. tropicalis* (Figure 5D-F). All above demonstrated that *C. tropicalis* promotes CRC progression through downregulation of PD-1 expression rather than PD-L1.

### Induction of autophagy by *C. tropicalis* mediated the regulation of PD-1 in SW480 cells

As we found that decreased tumor cell-intrinsic PD-1 expression mediated the CRC progression promoted by *C. tropicalis*, we then explored the mechanism by which *C. tropicalis* down-regulated the expression of PD-1 in SW480 cells. Studies have proved that autophagy can inhibit tumor genesis as tumor do not form, however, it also can provide energy and nutrition for tumor cell to promote tumor growth in progress cancers 21-23. In addition, previous study has already demonstrated that p62, as an important signaling adaptor involved in autophagy, is a multidomain protein which could activate the transcription factor NF-κB 24. It has been reported that NF-κB can regulate PD-1 expression in macrophages 25. In addition, autophagy is also a common defense method for cells when facing foreign microorganisms. Based on the above, we explored whether autophagy was participated in the CRC tumor growth induced by *C. tropicalis* and related to the down-regulation of PD-1. We firstly treated SW480 cells with *C. tropicalis* and performed autophagy functional assays. We found that co-culture with *C. tropicalis* up-regulated ATG7 and down-regulated p62 in the mRNA levels (Figure 6A). Similarly, increased LC3-II and ATG7 and decreased p62 expression in protein levels were detected in the co-cultured SW480 cells compared to the control SW480 cells (Figure 6B).

We established SW480 cells that stably expressed a tandem GFP-mCherry-LC3 construct. We found that *C. tropicalis* induced the autophagic flux in SW480 cells. Consistent with the *in vitro* study, we also found that the protein expression of LC3-II and ATG7 was increased while p62 was decreased in tumor tissues from *C. tropicalis* -treated CRC mice compared to control group (Figure 6D). These data suggested that *C. tropicalis* treatment can increase autophagy in SW480 cells. Furthermore, we treated SW480 cells with LY294002 and CQ respectively. The flow cytometry revealed that addition of LY294002 or CQ reversed the inhibition of tumor cell-intrinsic PD-1 expression in the *C. tropicalis*-cultured SW480 cells (Figure 6E, 6H). Similar results were found in the protein levels of PD-1 (Figure 6F, 6I). Correspondingly, compared to the *C. tropicalis* treatment alone, combination of LY294002 or CQ blocked the tumor cell growth, presenting as decreased cell proliferation (Figure 6G, 6J). Together, these data suggested that *C. tropicalis* down-regulated tumor cell-intrinsic PD-1 expression via induction of autophagy in SW480 cells.

### *C. tropicalis* inhibited PD-1 expression and promoted tumor growth through autophagy in CRC mouse xenograft model

**Figure 7:**
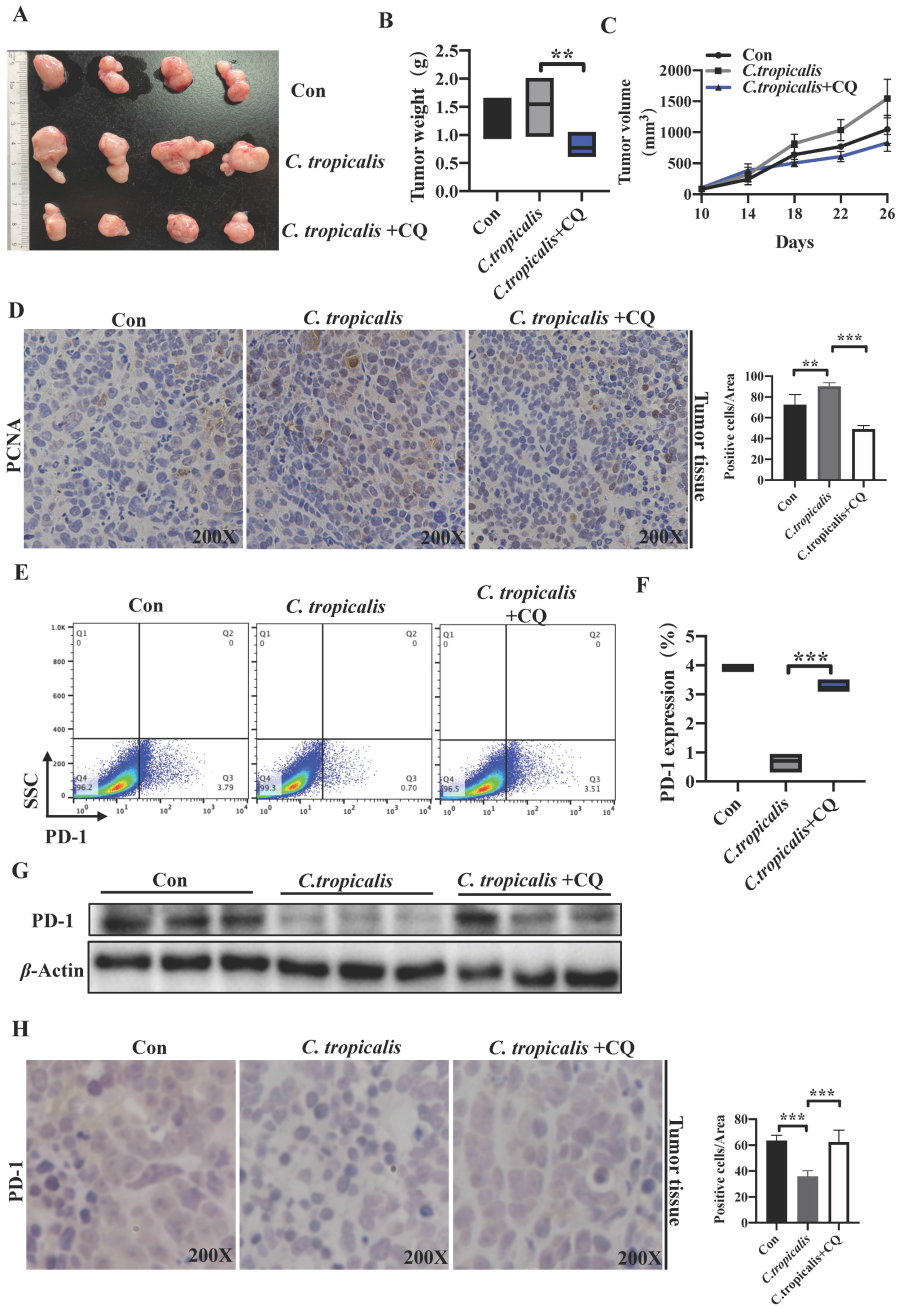
***C. tropicalis* inhibited PD-1 expression and promoted tumor growth through autophagy in CRC mouse xenograft model.** *C. tropicalis* and CQ were injected intratumorally and intraperitoneally respectively into the SW480 cells-induced xenograft CRC mouse model, n=4/group. Tumors were acquired. (A) Representative images of tumors from mice under different conditions. (B-C) Statistical analysis of tumor volumes and weights. (D) Representative images of immunohistochemical staining of tumors in different groups for PCNA. Positive cells of PCNA were counted using Image-Pro Plus software 6.0. (E-F) Tumor cell-intrinsic PD-1 expression in tumor tissues from different groups was detected by flow cytometry. Representative plots and analysis were shown. (G) Western blot was performed to determine the PD-1 protein expression in tumor tissues. Data with error bars are represented as mean ± SD. Each panel is a representative experiment of at least three independent biological replicates. *p < 0.05, **p < 0.01 and ***p<0.001 as determined by unpaired Student's t test.

To validate the observation that *C. tropicalis* down-regulated PD-1 expression and promoted CRC tumor growth is mediated by induction of autophagy *in vivo*. We established the SW480 cells-induced xenograft CRC mouse model and CQ (50 mg/kg) was injected intraperitoneally every other day for 3 weeks in the presence of *C. tropicalis.* We found that addition of CQ blocked the promoted effect of *C. tropicalis* on tumor growth, presenting as decreased tumor weight and volume (Figure 7A-C). Consistently, IHC staining showed less PCNA positive cells in tumors tissues from CQ and *C. tropicalis* co-treatment mice than those from *C. tropicalis*-treated mice (Figure 7D). Moreover, flow cytometry and western blot results confirmed that CQ also restored the expression of PD-1 in tumor tissues (Figure 7E-G). All above data suggested that *C. tropicalis* inhibited PD-1 expression and promoted CRC tumor growth through autophagy in xenograft CRC mouse model.

## Discussion

Previous studies have reported that PD-1 is mainly expressed on activated natural killer T cells, B cells, monocytes, and other immune cells 6, 10. The identification of PD-1 is always a coinhibitory receptor that functions as an immune checkpoint to induce immune tolerance 26, 27. However, many studies have proved that PD-1 is also expressed on melanoma tumor cells, hepatocellular carcinoma cells and NSCLC cells in recent years 11-14. Tumor cell-intrinsic PD-1 plays different roles in different cancers. The effect of PD-1 in melanoma and HCC is to promote tumor progression while in lung cancer PD-1 acts as an inhibitor.

Moreover, the function of PD-1 in immune cells depends on the regulation of downstream signals, including PI3K/AKT, MAPK/ERK1/2 and/or mTOR 9. Similarly, Shan Gao's team has proved that tumor cell-intrinsic PD-1 could inhibit tumor cell growth and the activation of AKT and ERK1/2 in NSCLC 14. Until now, no study focuses on whether PD-1 express on CRC tumor cells and explore the function of PD-1 in CRC tumor growth. Here, we determined that PD-1 is expressed in CRC cell lines. We also determined that CRC tumor cell-intrinsic PD-1 functions as a tumor suppressor independently of adaptive immunity. PD-1 knocked down or anti-PD-1 nivolumab treatment could promote CRC progression. Furthermore, we observed that PD-1 silenced increased the levels of AKT and ERK pathways. The above findings can explain the inadequate treatment effect of immunotherapy on CRC progression to a certain extent currently. This study suggests that we should take other therapies besides anti-PD-1 monoclonal antibody for CRC patients with high expression of tumor cell-intrinsic PD-1. This finding will benefit cancer patients via choosing the optimal immunotherapy strategies and improve the effectiveness of clinical cancer immunotherapy. Nonetheless, due to PD-1 signaling is always mediated via engagements of PD-L1 or PD-L2 ligands and tumor environments are complex, the mechanism by which tumor cell-intrinsic PD-1 promotes CRC tumor growth is not only limited to the AKT and ERK signals, more about the principles among this process need to be further investigated. Since all animal model experiments were performed on nude mice, the effect of tumor cell-intrinsic PD-1 on the tumor growth in the presence of adaptive immunity needs to be further explored using C57BL/6 mice.

More and more studies have shown that intestinal microbiota is participated in CRC progression 28, 29. Exposure to *Escherichia coli* carrying the colibactin-producing pks pathogenicity island directly resulted in a distinct mutation in CRC 30. *F. nucleatum* targets lncRNA ENO1-IT1 to promote glycolysis and oncogenesis in CRC 31. Other studies also found that *Bacteroides* can protect the intestinal mucosa and inhibit the occurrence of CRC 32, 33. All researches on the development of CRC promoted by intestinal commensal microorganisms are mostly focused on bacteria and few studies paid attention to commensal fungus. Our previous study has found *C. tropicalis* is specifically increased in Dectin3-/- colitis mice, CARD9-/- CRC mice and CRC patients 18, 34. In those researches we confirmed that *C. albicans* induced MDSC differentiation and activated MDSC function to promote CRC carcinogenesis. Furthermore, we found that *C. tropicalis* caused CRC chemotherapy resistance to oxaliplatin 19. Several studies have verified tumor cell-intrinsic PD-1 plays vital roles in cancers, no study has explored whether the CRC progression induced by *C. tropicalis* is related to PD-1 expressed on CRC tumor cells. In our work, *in vitro* and *in vivo* experiments indicated that high dose of *C. tropicalis* promoted CRC tumor growth. We also found that the expression of tumor cell-intrinsic PD-1 was decreased in *C. tropicalis* -cocultured SW480 cells. Meanwhile, we verified that the tumor cell growth was inhibited in* PDCD1*-overexpressed SW480 cells despite treatment with *C. tropicalis.* These suggested that *C. tropicalis* could down-regulate PD-1 expression to promote tumor growth. These findings supply a new mechanism by which *C. tropicalis* promotes CRC carcinogenesis and remind us the importance of monitoring the burden of *C. tropicalis*. We do not deny the role of other commensal microbiota in CRC progression, but these need to be further clarified.

It has been reported that NF-κB can regulate PD-1 expression in macrophages 25. Studies also proved that p62, as a vital substrate involved in autophagy, could activate the transcription factor NF-κB. So, we wanted to illustrate whether *C. tropicalis* down-regulated the expression of PD-1 in SW480 cells through autophagy. Here, we stimulated SW480 cells with *C. tropicalis* and autophagy inhibitor. We found that *C. tropicalis* on the one hand enhanced the flux of autophagy and promoted tumor growth in SW480 cells, on the other hand, it down-regulated PD-1 expression. However, autophagy inhibitor could block this facilitation and reverse the decreased expression of PD-1. Thus, our study clearly establishes that* C. tropicalis* inhibited PD-1 expression and promoted tumor growth through autophagy. The down-regulation of PD-1 expression by *C. tropicalis* through autophagy is only one aspect, and more about the mechanism of *C. tropicalis* regulating PD-1 needs to be further explored. Furthermore, the specific roles of p62 on this regulation also need our further investigation. In our further experiments, we will detect the expression of PD-1 and the level of autophagy in the tumor tissues of CRC patients, and analyze the correlation between *C. tropicalis*, PD-1 and autophagy in clinical samples.

In conclusion, our data show that PD-1 is expressed in CRC tumor cells in mRNA and protein levels. The tumor cell-intrinsic PD-1 suppresses the tumor growth independently adaptive immunity and inhibits the AKT and ERK1/2 signaling pathways. Knocking down PD-1 or anti-PD-1 antibody treatment can promote CRC progression. High dose of *C. tropicalis* down-regulates the expression of tumor cell-intrinsic PD-1 through autophagy to promote CRC tumor growth. This study provides a potential explanation for antibody mediated resistance. This reminds us that the detection of *C. tropicalis* burden, PD-1 expression and autophagy flux in tumor tissues from CRC patients are crucial before choosing immunotherapy strategies. It can benefit cancer patients and improve the effectiveness of clinical cancer immunotherapy.

## Supplementary Material

Supplementary figure.

## Figures and Tables

**Table 1:** Gene sequences

Gene	Sense (5'-3')	Anti-sense (3'-5')
*PDCD1*	CAGCTTGTCCAACTGGTCG	GCTCAAACCATTACAGAAGGCG
*PDCD1LG1*	GCTCCAAAGGACTTGTACGTG	TGATCTGAAGGGCAGCATTTC
*P62*	GCACCCCAATGTGATCTGC	CGCTACACAAGTCGTAGTCTGG
*ATG7*	CAGTTTGCCCCTTTTAGTAGTGC	CCAGCCGATACTCGTTCAGC
*ATG12*	CTGCTGGCGACACCAAGAAA	CGTGTTCGCTCTACTGCCC
*ATG16L1*	AACGCTGTGCAGTTCAGTCC	AGCTGCTAAGAGGTAAGATCCA
*β-actin*	CATGTACGTTGCTATCCAGGC	CTCCTTAATGTCACGCACGAT
