# Supplementary material for: C. tropicalis promotes CRC by down-regulating tumor cell-intrinsic PD-1 receptor via autophagy
Source: J Cancer. 2023 Jun 19;14(10):1794–808. doi: 10.7150/jca.79664 (PMC10355211; doi:10.7150/jca.79664)

Supplemental figure legends

Figure S1 PD-L1 inhibits tumor cell growth and activation of AKT and ERK1/2

(A) qRT-PCR expression analysis of *PDCD1LG1* mRNA levels in SW480 cells. (B-F) *PDCD1LG1* was silenced by siRNA in SW480 cells. The knockdown function of *PDCD1LG1* siRNA was verified by testing mRNA (B) and protein levels of PD-1 (C). CCK8 was used to detect the cell viability (D). Cell proliferation was determined by CFSE assay, quantification data was shown as MFI (n=3) (E). Western blot was used to analyze the expression of indicated proteins including p-AKT and p-ERK in cells transfected with the si*PDCD1LG1* (F). Data with error bars are represented as mean ± SD. Each panel is a representative experiment of at least three independent biological replicates. *$p < 0.05$, **$p < 0.01$ and ***$p<0.001$ as determined by unpaired Student's t test.

Supplemental Figure 1 PD-L1 inhibits tumor cell growth and activation of AKT and ERK1/2

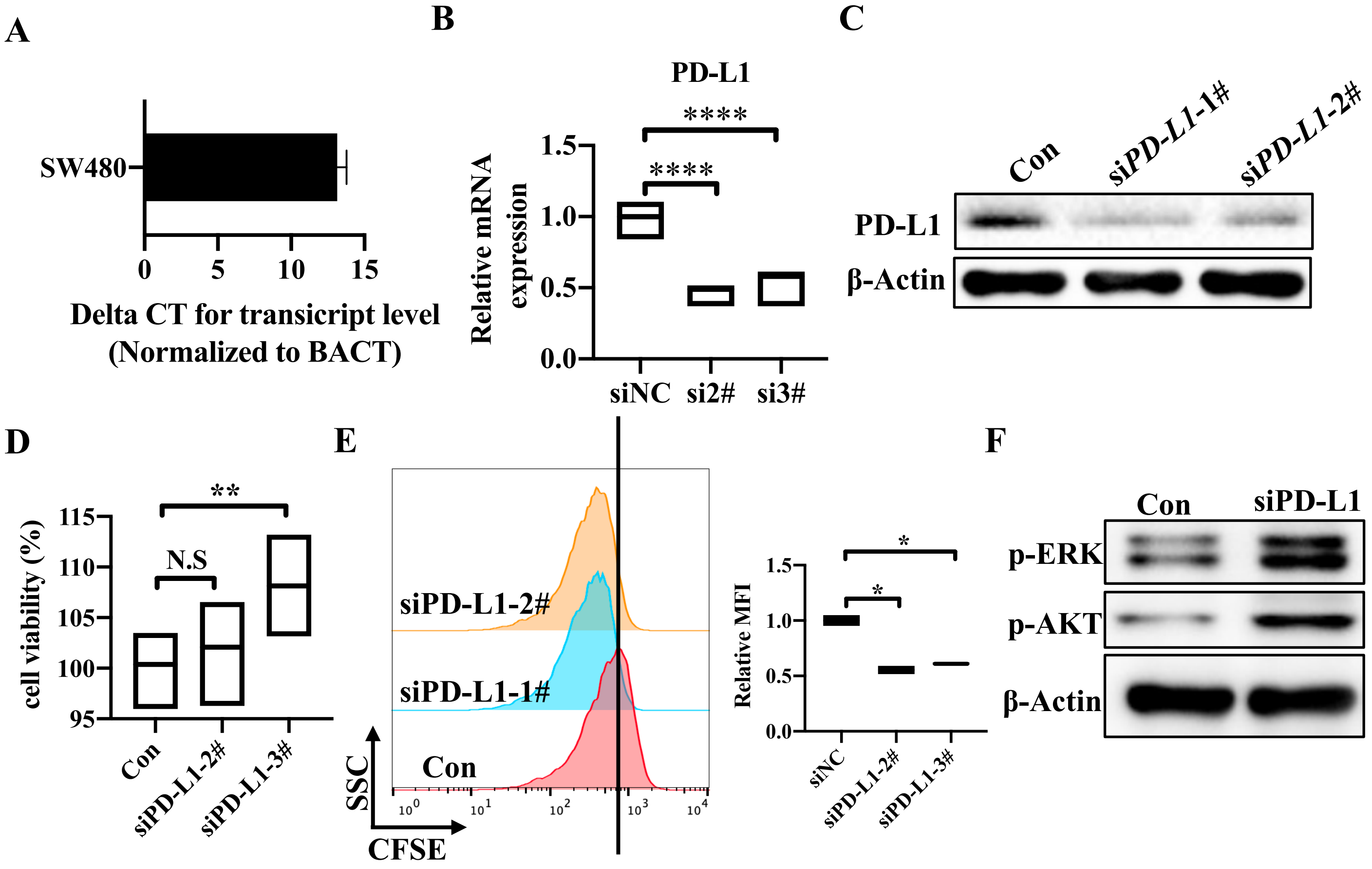

Supplement: Supplementary file 1 — Supplementary figure. [file jcav14p1794s1.pdf]
